# Circulating Antibodies to IDO/THO Pathway Metabolites in Alzheimer's Disease

**DOI:** 10.4061/2010/501541

**Published:** 2010-03-15

**Authors:** S. Duleu, A. Mangas, F. Sevin, B. Veyret, A. Bessede, M. Geffard

**Affiliations:** ^1^IDRPHT, 33400 Talence, France; ^2^Institute of Neuroscience of Castilla y León, Laboratory 14, 37007 Salamanca, Spain; ^3^Laboratoire IMS, EPHE-ENSCPB, Pessac, France

## Abstract

In Alzheimer's disease, indoleamine 2,3-dioxygenase and tryptophan hydroxylase are known to induce an overproduction of neurotoxic compounds, such as quinolinic acid and 3-hydroxykynurenine from the former, and 5-hydroxytryptophol and 5-methoxytryptophol from the latter. Other compounds, such as kynurenic acid, serotonin, and melatonin are produced via the same pathways. An improved ELISA method identified circulating antibodies directed against these compounds, linked to proteins, as previously described for other chronic diseases. This describes how only the A isotype of circulating immunoglobulins recognized a pattern of conjugated tryptophan metabolites in the sera of Alzheimer patients. These data indirectly confirmed the involvement of tryptophan derivatives in the pathogenic processes of Alzheimer's disease. Further studies are required to evaluate the relevance of these antibody patterns in monitoring this disease.

## 1. Introduction

Alzheimer's disease (AD) is a neurodegenerative disorder, resulting in a gradual, irreversible loss of memory and cognitive functions [1], mainly affecting cholinergic neurons. The severity of AD depends on the dysfunction of two molecules: Amyloid protein precursor (APP) and Tau protein. The aggregation of these proteins results in senile plaque formation and neurofibrillar degeneration. Pathological mutations have been discovered on the APP gene, in the region coding for the Beta amyloid peptide (A*β*), as well as on the presenilin PS1 and PS2 genes. PS1 and PS2 proteins regulate APP catabolism. Despite this new knowledge, the etiology of AD remains largely unknown. Common pathogenic disorders reported in AD include: autoimmunity [2], excitotoxicity, and oxidative and radical processes [3], all inducing neuron death and the activation of microglia cells and astrocytes [4].

**Figure 1:**
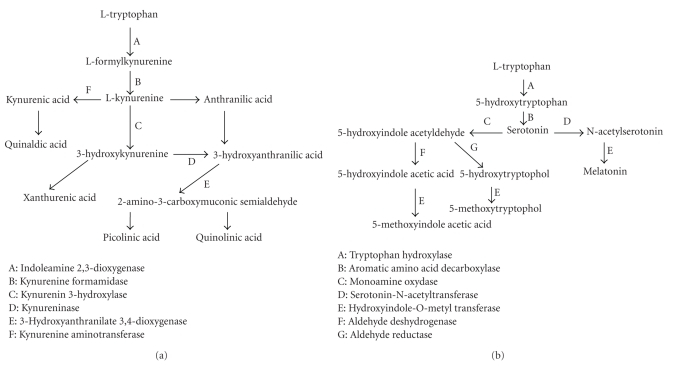
(a) The IDO-1 pathway. (b) the THO pathway.

One mechanistic hypothesis focuses on the tryptophan molecule, an amino acid essential for cell growth and metabolism. In the central nervous system, tryptophan is metabolized via two pivotal biochemical pathways [5], shown in Figures 1(a) and 1(b).

In the first pathway, tryptophan is metabolized by indoleamine-2, 3-dioxygenase-1 (IDO-1), an enzyme found in many tissues. IDO-1 catalyses tryptophan to *N*-formylkynurenine, an intermediate for several biochemical compounds. Moreover, IDO-1 is an inducible enzyme, activated in AD by proinflammatory cytokines, such as interferon-gamma (IFN-*γ*) [6], interleukin-12 (IL-12), interleukin-18 (IL-18) [7], and the A*β* 1-42 fragment [8]. Tryptophan catabolism abnormalities have been observed in AD. The tryptophan catabolism [9] and seric kynurenine/tryptophan ratio [10] increase in AD patients. Neuroinflammation in the central nervous system (CNS) may be a major factor in this disease, due to cytotoxic tryptophan metabolite production by CNS infiltrating macrophages and glial cells [11]. Dementia in AD patients is correlated with the overproduction of quinolinic acid (Quina) [12, 13], a metabolite of tryptophan accumulated in neurons and astrocytes via proinflammatory processes [14].

In the second pathway, tryptophan hydroxylase (THO), a rate-limiting enzyme, generates serotonin (5-HT) and melatonin (Mel), among other compounds. A loss of serotoninergic neurons has also been observed [15]. Mel is an important antioxidant, anti-inflammatory mediator [16] that interacts directly with A*β* deposition and hyperphosphorylated Tau protein. It plays a role in cholinergic neuroprotection [17]. THO activity decreases in the aging brain [18]. However, the THO pathway indirectly produces neurotoxic metabolites, such as 5-methoxytryptophol (5-MTol), 5-hydroxytryptophol (5-HTol), and the oxidative compound 5-hydroxyindole acetic acid (5-HIAA) via an enzyme cascade. All of these molecules play numerous roles in AD [19].

The aim of this study was to assay circulating antibodies directed against tryptophan derivatives conjugated to proteins in order to mimic the pathogenic mechanisms in vivo. The antibody titers in AD patient sera were thus compared to controls. The identification of specific antibodies in AD may enhances our understanding of some of the immunological processes involved.

## 2. Materials and Methods

### 2.1. Patient Sera

The study was conducted in accordance with Good Clinical Practice guidelines, with the informed consent of the patients, their caregivers, and the controls, in application of French and European law and current medical procedures. In this study, healthy control populations were matched by age and sex with the AD patients. Serum samples from 48 patients (age range: 65–85) were used. There was no subclassification among the AD states associated with dementia. Twenty serum samples were obtained from healthy controls (age range: 64–82). 

AD was diagnosed according to the criteria outlined by the National Institute of Neurological and Communicative Disorders and Alzheimer's Disease and Related Disorders Association (NINCDS-ADRDA), in the absence of any clinical or laboratory evidence of a cause other than AD for dementia [20]. The patients had mild to moderately severe disease as defined by the Mini-Mental State Examination (MMSE) [21], with scores of 10 to 26, and screening and baseline Clinical Dementia Rating (CDR), with scores of 1 or 2 [22]. None of the patients had AD aggravated by an additional diagnosis of delusion, delirium, or depression, and none had a known or suspected history of alcoholism or drug abuse.

### 2.2. Conjugate Synthesis

Each tryptophan derivative was dissolved in 200 *μ*L dimethylsulfoxide (DMSO) (Acros). Bovine serum albumin (BSA) (ID Bio) was also dissolved in 3 mL 2-morpholino-ethanesulfonic acid monohydrate (MES) buffer 10^−1^ M (pH 6.3) (Acros). Then, the tryptophan derivatives were mixed with the BSA solution and supplemented with 15 mg N-hydroxysuccinimide (Sigma) and 1-(3-dimethylaminopropyl)-3-ethylcarbodiimide (Acros) as coupling agents [23]. The conjugates were synthesized by linking 10 mg kynurenine (Kyn) (Sigma), or 3-hydroxykynurenine (3-OHKyn) (Sigma), kynurenic acid (Kyna) (Acros), Quina (Acros), quinaldic acid (Quinald) (Acros), 3-hydroxyanthranilic acid (3-OHAnthra) (Aldrich), anthranilic acid (Anthra) (Acros), xanthurenic acid (Xantha) (Acros), picolinic acid (Pico) (Acros), or 5-hydroxyindole acetic acid (5-HIAA) (Sigma), to 20 mg BSA. The coupling reaction took place in darkness, at 37°C, for 1 hour. The reaction was stopped by adding 100 mg hydroxylamine (Sigma-Aldrich) per conjugate. The protein conjugates were dialyzed with 10^−1^ M NaCl solution for 72 hours and the bath solution was changed at least four times per day. The conjugated tryptophan derivative and BSA concentrations were evaluated by spectrophotometry. The coupling ratio of each conjugate was calculated from the absorbance values. 

Tryptophan (Sigma), 5-hydroxytryptophan (5-HW) (Sigma), and 5HT (Sigma) were linked to BSA with glutaraldehyde (G), as previously described in [24, 25]. 

Mel (Sigma), 5-MTol (Sigma), and 5-HTol (Sigma) conjugates were synthesized as follows: 5 mg of each hapten were shaken in 1 mL water/ethanol (vol/vol) and mixed with 20 mg BSA (previously dissolved in 1 mL deionized water) plus 600 *μ*L 3M acetate buffer (Sigma). One mL 2% formaldehyde solution was added to the mixture and the reaction was stabilized after 5 minutes at room temperature. The conjugates were dialyzed with a 10^−2^ M phosphate buffer solution (NaH_2_PO_4_, 12H_2_O) and 0.15M NaCl, (pH 7.4), 3 times per day for 72 hours.

### 2.3. Evaluation of the Molecular Coupling Ratio

The molecular coupling ratio of each conjugate was determined by measuring the concentration of tryptophan derivative and BSA at 310–330 nm and 280 nm, respectively, as previously described in [23], taking into account the molar extinction coefficients after coupling.

### 2.4. ELISA

ELISA was used to determine the titers of G, M, or A immunoglobulins (Ig). The protocol has been extensively described elsewhere [23–25]. Briefly, polystyrene 96-well plates (NUNC) were coated with 200 *μ*L solution containing 10–50 *μ*g/mL tryptophan-derivative conjugates in 0.05 M carbonate buffer (pH 9.6). Well plates were incubated under agitation at 4°C for 16 hours. Then, 200 *μ*L blocking buffer A (PBS, 2.5 g/L BSA) were applied and samples were incubated at 37°C for 1 hour. Well plates were washed with PBS solution and filled with 200 *μ*L serum diluted 1:500 in blocking buffer A for IDO derivatives and 5-HIAA or blocking buffer B (PBS, 10% glycerol and 2.5 g/L BSA) for the other conjugates. They were incubated at 37°C for 2 hours. Well plates were washed 3 times with PBS, 0.05% Tween 20, incubated with peroxidase-labeled antihuman IgG (Biorad), anti-human IgM (Pierce), or anti-human IgA (Pierce) antibodies at 37°C for 1 hour. These anti-isotype antibodies were diluted 1: 50,000, 1: 25,000, and 1: 14,000 in blocking buffer C (PBS, 0.05% Tween 20, 2.5 g/l BSA), respectively. Plates were then washed three times with PBS, 0.05% Tween 20, and incubated with the detection solution in darkness for 10 minutes. The chromogen solution consisted of 8% orthophenyldiamine (OPD, Sigma Aldrich) in a 0.1, M sodium citrate and 0.01, M phosphate buffer (pH 5.0), containing 0.01% H_2_O_2_ (Merck) for the peroxidase assay. The reaction was stopped using 50 *μ*L 2-N HCl (Sigma-Aldrich). Optical densities (ODs) were measured at 492 nm using a Multiscan spectrophotometer. All assays were carried out in duplicate.

### 2.5. Statistical Analysis

The OD of each BSA-coated well was subtracted from the OD of each well containing the tryptophan derivative. The Mann and Whitney U-test was used to compare the AD and healthy sera for each tryptophan-derivative conjugate. All statistical analyses were considered significant when *P* ≤ .01. The proportion of positive sera was calculated as the number of patients with an OD above the mean control group OD value +2 standard deviations.

## 3. Results and Discussion

**Figure 2:**
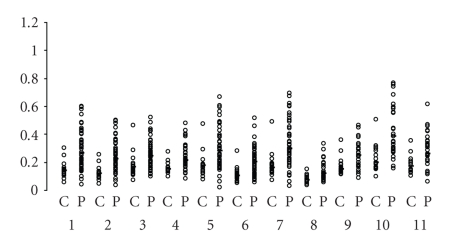
Scattergrams represent the OD values for AD patients (P) and controls (C) for each conjugate (statistically significant) and isotype (statistically significant). The first lane of OD values corresponds to C and the second to P. IgA for: (1) 3-OHKyn, (2) Kyna, (3) Quina, (4) 3-OHAnthra, (5) Anthra, (6) Xantha, (7) Pico, (8) 5-HT, (9) Mel, (10) 5-HIAA, and (11) 5-MTol.

The presence of circulating antibodies directed against conjugated tryptophan metabolites indirectly revealed the overproduction of metabolites associated with hyperactivation of the IDO-1 in AD, as previously described in [23]. However, no previous study had shown the presence of circulating antibodies against THO-pathway-derived metabolites. Some statistically significant results are shown in Figure 2 and Table 1. IgA responses were observed only for the antibodies directed against the following IDO-1 pathway metabolites: 3-OHKyn, Kyna, Quina, 3-OHAnthra, Anthra, Xantha, and Pico. The role of Quina and 3OH-Kyn in neurological disorders has been previously described in numerous studies [26]. An accumulation of Quina in astrocytes and neurons is one of the events associated with depression or dementia in AD. Quina acts as an agonist of the N-methyl-d-aspartate (NMDA) receptor and plays a direct role as an excitotoxic agent [27]. Rahman et al. [28] showed that Quina was colocalized with the hyperphosphorylated Tau protein of cortical neurons in AD brains and induced Tau protein phosphorylation. Decreased concentrations of Kyna, a Quina antagonist, were found in AD patient sera: Hartai et al. [29] reported a decrease in Kyna concentrations in plasma and red-blood cells, while Kyn levels and kynurenine aminotransferase I and II activity remained unchanged.

However, our results revealed antibodies directed against conjugated Kyna in the sera. Moreover, Baran et al. [30] previously observed that a significant increase in Kyna production in the putamen and caudate nucleus of AD patients was associated with an elevated kynurenine metabolism. Xantha production via the IDO-1 pathway is higher in depressed patients than controls [31] and plays a role in apoptosis [32], as well as acting as a neuromodulator in the rat brain [33]. The production of 3-OH anthra, Anthra, and Pico in AD had not previously been studied in sufficient detail. The IDO-1 pathway is a key regulator of the immune response. IDO-1 induction and expression tends to limit the extracellular tryptophan pool necessary for lymphocyte proliferation [34] and pathogen invasion [35].

As in the case of the IDO-1 pathway, IgA antibodies were also found against some THO pathway metabolites, that is: the neurotransmitter 5-HT, the neuroprotector Mel, and the neurotoxic metabolites 5-HIAA, and 5-MTol. Mel and 5-MTol production is dependent on the overexpression of Hydroxyindole-O-metyl transferase, which may be indirectly responsible for the large increase in circulating antibodies directed against Mel and 5-MTol, synthesized mainly in the pineal gland [36]. Their production obeys a circadian rhythm in healthy persons, which tends to disappear in AD patients [37].

Burke et al. [38] reported that, in AD, 5-HT and 5-HIAA production was specifically localized in the raphe nucleus. A decrease in THO transport to axon terminals resulted in increased concentrations of these molecules, as well as a 4.7-fold increase in THO activity. High 5-HIAA levels have been measured in the delirious phase of AD [39].

A major finding in this work is that all the circulating antibodies detected were of the IgA isotype, associated with mucosal immunity, stimulated by exogenous factors (e.g., bacteria constituents). Along those lines, Malaguarnera et al. [40] demonstrated a correlation between the levels of circulating antibodies directed against *Helicobacter pylori* antigens and AD scores. Many authors have suggested that bacteria play a role in the etiology of AD [41]. Miklossy et al. demonstrated in vitro that exposing neuronal and glial cells to *Borrelia* spirochetes induced morphological changes related to amyloid deposition, similar to those observed in AD [42]. Moreover, IDO-1 is induced by many interleukins and gram-bacteria lipopolysaccharides [43]. IDO-1 is considered an immunomodulator, as tryptophan “starvation” prevents bacterial multiplication [44]. Moreover, the overproduction of IDO-1 pathway metabolites is linked to many cell processes associated with inflammation and apoptosis [45]. Further investigations should thus focus on IDO-1 and THO derivatives linked to endogenous proteins and/or bacteria components.

## 4. Conclusion

Circulating antibodies, exclusively of the IgA isotype, directed against tryptophan metabolites were found in AD patient sera, thus demonstrating that neurotoxic tryptophan metabolites are involved in this neurodegenerative disease. Activation of the IDO-1 pathway leads to overexpression of these tryptophan metabolites. The production of IgA antibodies suggests the activation of the mucosal immune system, possibly by bacterial components. The sequence of events may start when circulating bacteria components induce IDO-1 activity. The identification of circulating antibodies directed against IDO-1/THO pathway metabolites contributes to elucidating the etiology of AD.

## Figures and Tables

**Table 1:** Significance (*P* ≤ .01) of OD values (U Mann and Whitney *t*-test) and percentage of AD positive patients on well plates coated with tryptophan-derivative conjugates. OD values for each tryptophan-derivative conjugate were subtracted from those on BSA well plates. Percentages were calculated as follows: number of patients with OD values above mean OD value of controls +2 standard deviations.

		Conjugates coated on well plates							
Isotype		Kyn	3-0HKyn	Kyna	Quina	Quinald	3-OHAnthra	Anthra	Xantha
G	Significance	0.0836	0.5746	0.5758	0.8793	0.7513	0.0194	0.1576	0.0635
	Percentage	25.4%	10.9%	10.9%	7.2%	16.3%	9.0%	14.7%	20.0%
M	Significance	0.8275	0.8741	0.9784	0.0000	0.3450	0.2294	0.8172	0.9895
	Percentage	29%	9.0%	18.1%	7.2%	32.7%	14.5%	21.8%	21.8%
A	Significance	0.0015	**0.0001**	**0.0000**	**0.0027**	0.0119	**0.0034**	**0.0026**	**0.0005**
	Percentage	38.1%	**58.1%**	**61.8%**	**65.4%**	43.6%	**50.9%**	**54.5%**	**63.6%**

Isotype		Pico	W	5-HW	5-HT	Mel	5-HIAA	5-HTol	5-MTol

G	Significance	0.0790	0.9042	0.0000	0.0118	0.3873	0.0878	0.4244	0.6936
	Percentage	12.7%	24.1%	27.5%	20.6%	20.6%	10.3%	10.3%	6.8%
M	Significance	0.9737	0.0007	0.0000	0.7928	0.4906	0.4181	0.3696	0.6941
	Percentage	23.6%	3.4%	3.4%	10.3%	10.3%	24.1%	6.8%	3.4%
A	Significance	**0.0011**	0.0878	0.2836	**0.0001**	**0.0018**	**0.0001**	0.0010	**0.0015**
	Percentage	**65.4%**	10.3%	13.7%	**62%**	**51.7%**	**58.6%**	41.3%	**51.7%**
